# A Novel Risk Model for lncRNAs Associated with Oxidative Stress Predicts Prognosis of Bladder Cancer

**DOI:** 10.1155/2022/8408328

**Published:** 2022-10-11

**Authors:** Lixiang Feng, Kang Yang, Qihui Kuang, Min Peng, Lili Li, Pengcheng Luo

**Affiliations:** ^1^Department of Urology, Wuhan Third Hospital, School of Medicine, Wuhan University of Science and Technology, Wuhan 430060, China; ^2^Department of Urology, Renmin Hospital of Wuhan University, Wuhan 430060, China; ^3^Department of Oncology, Renmin Hospital of Wuhan University, Wuhan 430060, China; ^4^Central Laboratory, Renmin Hospital of Wuhan University, Wuhan 430060, China

## Abstract

**Background:**

Oxidative stress (OS) reactions are closely related to the development and progression of bladder cancer (BCa). This project aimed to identify new potential biomarkers to predict the prognosis of BCa and improve immunotherapy.

**Methods:**

We downloaded transcriptomic information and clinical data on BCa from The Cancer Genome Atlas (TCGA). Screening for OS genes was statistically different between tumor and adjacent normal tissue. A coexpression analysis between lncRNAs and differentially expressed OS genes was performed to identify OS-related lncRNAs. Then, differentially expressed oxidative stress lncRNAs (DEOSlncRNAs) between tumors and normal tissues were identified. Univariate/multivariate Cox regression analysis was performed to select the lncRNAs for risk assessment. LASSO analysis was conducted to establish a prognostic model. The prognostic risk model could accurately predict BCa patient prognosis and reveal a close correlation with clinicopathological features. We analyzed the principal component analysis (PCA), immune microenvironment, and half-maximal inhibitory concentration (IC50) in the risk groups.

**Results:**

We constructed a model containing eight DEOSlncRNAs (AC021321.1, AC068196.1, AC008750.1, SETBP1-DT, AL590617.2, THUMPD3-AS1, AC112721.1, and NR4A1AS). The prognostic risk model showed better results in predicting the prognosis of BCa patients and was strongly correlated with clinicopathological characteristics. We found great agreement between the calibration plots and prognostic predictions in this model. The areas under the receiver operating characteristic (ROC) curve (AUCs) at 1, 3, and 5 years were 0.792, 0.804, and 0.843, respectively. This model also showed good predictive ability regarding the tumor microenvironment and tumor mutation burden. In addition, the high-risk group was more sensitive to eight therapeutic agents, and the low-risk group was more responsive to five therapeutic agents. Sixteen immune checkpoints were significantly different between the two risk groups.

**Conclusion:**

Our eight DEOSlncRNA risk models provide new insights into predicting prognosis and clinical progression in BCa patients.

## 1. Introduction

Bladder cancer (BCa) is a tumor growing in the mucosa of the bladder and is one of the most prevalent urogenital malignancies, ranking sixth among male-related cancers [1]. In today's clinical treatment, cystoscopy pathological biopsy is the gold standard for detecting this highly heterogeneous cancer [2, 3]. BCa is categorized into two categories based on tumor invasion in the bladder: nonmuscle-invasive and muscle-invasive. Nonmuscle-invasive bladder cancer (NMIBC) accounts for almost 70% of all newly diagnosed BCa patients [4]. Tumor excision is the most common therapy for NMIBC, followed by immunotherapy with intravesical BCG vaccine or intravesical chemotherapy [5]. The 5-year survival rate of NMIBC patients accounts for about 90% of all BCa patients, with a postoperative recurrence risk of 50% to 70% [6]. Nearly 25% of NMIBC patients eventually develop muscle-invasive bladder cancer (MIBC), which may be related to drug resistance [7]. Although the current combination of surgical, radiotherapy, chemotherapy, and targeted treatment regimens have extended overall patient survival to some extent, the overall patient recurrence and mortality rates of BCa are still high [5]. Due to the high heterogeneity of BCa, personalized medicine is an excellent technique to increase therapy outcomes and patient prognosis. Personalized medicine necessitates a diverse set of proven molecular biomarkers, including early diagnostic and prognostic indicators that can assist doctors in identifying patients in need of early aggressive therapy and predicting patients' responses to developing targeted medicines [8]. In the last several years, immunotherapy using immune checkpoint inhibitors has been gradually utilized for treatment and achieved some efficacy. This approach includes anti-PD-L1 therapies, atezolizumab, avelumab, nivolumab, and pembrolizumab, which are approved only in the metastatic stage [9–11]. However, relatively few biomarkers are used to evaluate immunotherapy's efficacy in BCa. As a result, new and precise efficacy assessments in BCa therapy are urgently needed.

Oxidative stress (OS) was originally defined in 1985 as a state of imbalance between oxidative and antioxidant activities in the body that favors oxidation and leads to inflammatory infiltration of neutrophils and increased release of proteases. Production of numerous oxidative intermediates in aging and disease development kick in [12]. Free radicals generated by redox processes, such as ROS and other compounds with unpaired electrons, can damage DNA, proteins, and lipids, causing tissue damage [13]. ROS can alter TGF-*β*1-induced ECM synthesis through the p38MAPK/Akt signaling pathway [14]. The ECM is an essential part of the tumor microenvironment, and tumors can also control the ECM to induce ROS generation under pathological conditions. In normal tissues, ROS may maintain a normal range with ECM, but under pathological conditions, this standard range will be broken, and the underlying mechanism needs more exploration to explain [15]. OS in cellular physiology is caused by an imbalance between reactive oxygen species (ROS) and antioxidant signaling. This shows that ROS is a critical component of the OS. There is abundant evidence that the continuous production of ROS in the body can promote the survival of cancer cells [16, 17]. They can trigger oncogenic signals, increase cell survival and proliferation, and cause DNA damage and genetic instability. On the other hand, as the tumor progresses, the level of ROS gradually increases. When the level of ROS is higher than the redox threshold of the tumor microenvironment, the antitumor signal is generated, which starts to induce tumor cell death and affects the relationship between tumor and normal cells. The redox balance between ROS suggests that ROS can also be a target for tumor therapy. Thus, OS has a dual purpose in cancer cell physiology [18–20]. Many lncRNAs may be regulated by OS during carcinogenesis, and lncRNAs could modulate OS by enhancing or inhibiting the oxidative/antioxidant system [21]. ROS-related lncRNAs may not only act as direct biomarkers to assist in differentiating patients with cancer but also help clinicians monitor dynamic redox homeostasis and assess the risk of developing cancers. Therefore, developing additional ROS-related lncRNA biomarkers is crucial for the early diagnosis of cancers [21].

In recent years, the rise of big biological data mining for patient prognosis has driven the rapid development of personalized medicine [22]. Previous studies have analyzed OS genes and constructed prognostic models for various tumor types, including gastric cancer [23]. However, most studies on traditional lncRNAs have been still in their early stage. Therefore, it is imaginable that lncRNA-based therapies in clinical studies were imminent [24]. Several lncRNAs associated with OS have been proven essential to tumor initiation and progression [24]. lncRNA MALAT1 increased ROS levels and promoted head and neck squamous cell carcinoma metastasis [25]. Vascular endothelial growth factor A (VEGFA) had worsened OS and increased cancer development [26]. Another carcinogenic lncRNA, LINC00173.v1, had been found to induce angiogenesis and the development of lung squamous cell carcinoma. In vivo studies showed that antisense oligonucleotides specific for LINC00173.v1 had a better anticancer effect and enhanced the sensitivity of lung squamous cell carcinoma to cisplatin [27].

This research focused on exploring differentially expressed oxidative stress lncRNAs (DEOSlncRNAs) in BCa and assessed their prognostic significance. RNA-sequencing and somatic mutation data in Bca patients were obtained from The Cancer Genome Atlas (TCGA) database. In this research, we identified eight OS-related lncRNAs to construct the prognostic risk model, which could enhance the prognostic prediction of BCa patients with various clinical circumstances. We further analyzed the differences in clinical characteristics and related prognoses through risk-prognosis groupings, a nomogram, functional enrichment analysis, tumor mutation burden analysis, and immune infiltration assessment. This model showed a foundation for further investigation of immune mechanisms, new therapeutic targets, and clinical agents.

## 2. Methods

### 2.1. Identification of Differentially Expressed OS-Related lncRNAs

In total, we collected 807 OS genes from the GeneCards database, and 172 differentially expressed OS genes were identified using the “Limma” package in the R project (log2|fold change (FC)| ≥1.0, and *p* < 0.05) [28]. A total of 14056 lncRNAs were abstracted from the TCGA database. Furthermore, Pearson correlation analysis identified a correlation analysis between 172 differentially expressed OS genes and 14056 lncRNAs. A total of 1157 OS-related lncRNAs (OSlncRNAs) were identified in BCa (|cor| >0.4, *p* < 0.001). Finally, 720 differentially expressed OSlncRNAs (DEOSlncRNAs) were attained by using the Limma package (log2|FC| ≥1.0 and *p* < 0.05).

### 2.2. Construction of the Prognostic Signature

396 BCa samples acquired from TCGA were randomly separated into a training cohort and a test cohort in a 1 : 1 ratio to create a lncRNA-based signature. By univariate Cox analysis, we identified potential DEOSlncRNAs that showed great prognostic value for BCa in the training cohort (*p* < 0.05). Then, we conducted a LASSO regression analysis to remove the overfitting variables. Subsequently, a DEOSlncRNA signature was further generated using multivariate Cox regression to analyze the hazard ratios of potential lncRNAs. The risk of DEOSlncRNA signature = exp (DEOSlncRNAs) × *β*, where *β* is the coefficient of each candidate DEOSlncRNA from the multivariate Cox analysis.

The lncRNA-mRNA coexpression network was used to display OS-related lncRNAs and their corresponding mRNAs in risk models. Cytoscape software (version 3.7.2) was utilized to visualize coexpression networks. By using the R studio software of the “ggalluvial” R package, Sankey plot were applied to reveal complex relationships [29].

### 2.3. Evaluation and Validation of the Risk Model

Risk scores were analyzed for each BCa patient. Based on the median risk score, all patients were classified into high-risk groups (high-risk score) and low-risk groups (low-risk score). The prognosis of the two risk groups was compared using KM survival curves. Time-dependent receiver operating characteristic (ROC) curve analysis was performed using the “survival,” “survminer,” and “timeROC” R packages [29], assessing prognostic feature specificity and sensitivity. The area measured prognostic accuracy under the ROC curve (AUC), a measure of discrimination. Principal component analysis (PCA) was performed using the “ggplot2” R package to explore distinguishability [29]. Afterward, the distribution of patient risk scores and scatter plots were plotted to visualize the detailed correlation of death status with risk scores.

A boxplot was utilized to assess the association of clinical features with risk scores. The two risk groups performed *p* value tests using KM survival curves to compare clinical characteristics and prognostic closure.

### 2.4. Construction of the Predictive Nomogram

The 396 cases with accompanying clinical data were employed for the univariate and multivariate Cox regression analysis. A nomogram constructed combining DEOSlncRNAs and other clinicopathological features was applied to predict prognostic outcomes in BCa patients. Calibration curves were created to validate the accuracy of the nomogram.

### 2.5. Gene Set and Functional Enrichment Analysis

The samples were separated into two groups based on the median risk estimates. The genes that differed in expression between the high- and low-risk groups were discovered with |log2FC| ≥ 1 and a false discovery rate (FDR) <0.05 using the R package “Limma.” Gene Ontology (GO) analysis and Kyoto Encyclopedia of Genes and Genomes (KEGG) pathway analysis were applied to explore the gene functions and identify the signaling pathways related to the differentially expressed genes.

### 2.6. Analysis of Tumor-Infiltrating Immunocyte and Immune Checkpoints

CIBERSORT, an immune-related algorithm that analyzes the abundance of 22 immunocyte types, was used to display the immune landscape of BCa samples. We compared the risk scores and immune checkpoint activation between the low- and high-risk groups by the R package “ggpubr” [30].

### 2.7. Estimation of Tumor Mutational Burden

Tumor mutational burden (TMB) was a novel therapeutic metric for determining immunotherapy sensitivity. The R package “maftools” [31] were used to handle somatic mutation data, which includes somatic coding, base replacement, and insert-deletion mutations. The median TMB value was utilized as the cutoff point for classifying BCa patients as high-TMB or low-TMB.

### 2.8. Exploration of the Model in Clinical Treatment

Derived from the Genomics of Drug Sensitivity in Cancer (GDSC) database, the R package “pRRophetic” [32] was performed to analyze the gene therapy response defined by the half-maximal inhibitory concentration (IC50) in each BCa patient.

### 2.9. Statistical Analysis

R version 4.1.2 was applied to examine all statistical data. Kaplan-Meier survival analysis was performed to detect survival distinctions between the two risk groups. Statistical analysis was performed using flexible statistical methods and was statistically significant when the *pvalue* was less than 0.05.

## 3. Results

### 3.1. Acquisition of Differentially Expressed OSlncRNAs

**Figure 1:**
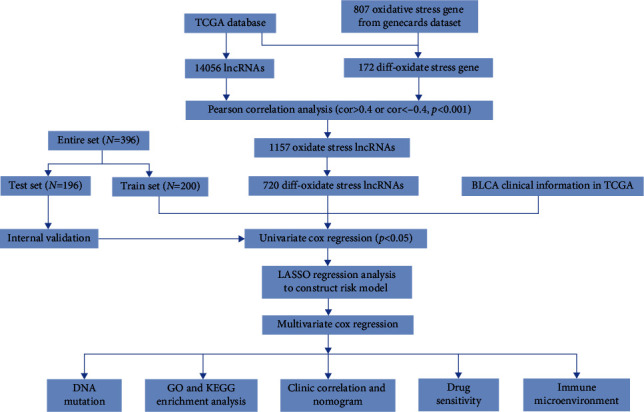
Study design and flowchart of this study.

**Figure 2:**
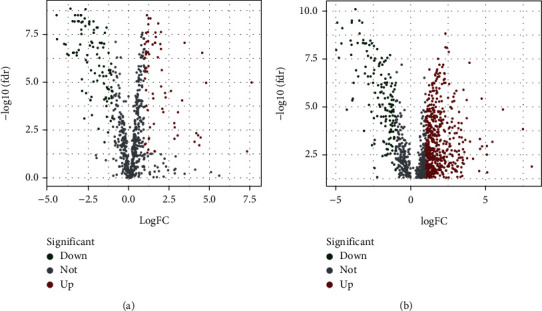
The volcano plot of differentially expressed oxidative stress related genes and lncRNAs. (a) The volcano plot of 172 differentially expressed oxidative stress genes extracted from GeneCards database. (b) The volcano plot of 720 differentially expressed oxidative stress-related lncRNAs (DEOSlncRNAs).

The flowchart of this study is shown in Figure 1. A total of 14056 lncRNAs were extracted from transcriptome data of BCa from TCGA. 807 OS-related genes were extracted from the GeneCards database, and 172 differentially expressed OS-related genes were identified, including 71 upregulated and 101 downregulated genes (Figure 2(a)). Then, the coexpression relationship was analyzed between 14056 lncRNAs and 172 differentially expressed OS-related genes. In total, 1157 lncRNAs were identified as OS-related lncRNAs (OSlncRNAs). Finally, 720 differentially expressed OSlncRNAs (DEOSlncRNAs) were identified (Figure 2(b)).

### 3.2. Development of a Prognostic Risk Model

**Figure 3:**
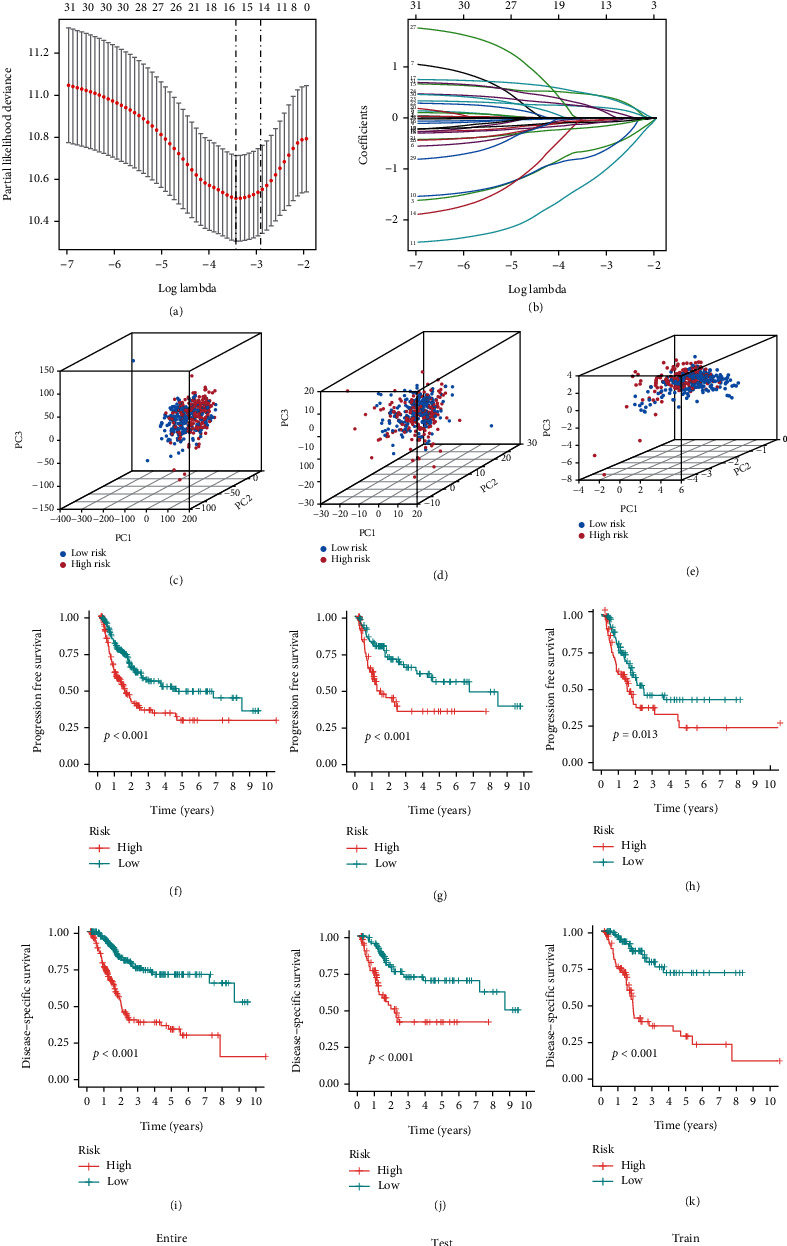
Construction of the prognostic risk model. (a) The LASSO coefficient profile of 8 differential oxidative stress lncRNAs. (b) The 10-fold cross-validation for variable selection in the LASSO regression. (c) PCA plot of risk score based on the expression profiles of all genes. (d) PCA plot of oxidative stress genes. (e) PCA plot of oxidative stress prognostic risk-related lncRNAs. (f–h) Kaplan–Meier curves of progression free survival between the high-risk and low-risk groups in the entire, test, and training sets. (i–k) Kaplan–Meier curves of disease-specific survival between the high-risk and low-risk groups in the entire, test, and training sets.

We integrated clinical characteristics from the BCa cohort in TCGA and excluded individuals with a survival duration of fewer than 30 days. A total of 396 patients were randomly allocated to the train and test groups. We identified 32 prognosis-associated DEOSlncRNAs in the train set through univariable Cox analysis. A multivariate analysis was then performed. Eight DEOSlncRNAs (AC021321.1, AC068196.1, AC008750.1, SETBP1-DT, AL590617.2, THUMPD3-AS1, AC112721.1, and NR4A1AS) were identified to develop a risk model owing to the coefficient as a result of the LASSO Cox regression and multivariate analyses (Figures 3(a) and 3(b)). The risk score = [AC021321.1 × (−1.0265)] + [AC068196.1 × (−1.8010)] + [AC008750.1 × (−2.0138)] + [SETBP1 − DT × (0.7344)] + [AL590617.2 × (0.6131)] + [THUMPD3 − AS1 × (−0.3366)] + [AC112721.1 × (−0.3353)] + [NR4A1AS × (0.3252)]. This indicated that AC021321.1, AC068196.1, AC008750.1, and THUMPD3-AS1 were lowly expressed in the high-risk group, and SETBP1-DT, AL590617.2, AC112721.1, and NR4A1AS were highly expressed in the high-risk group (Table 1). We showed the chromosomal location, transcript length, and subcellular localization of eight lncRNAs by using the DIANA Tools. Among them, AC068196.1 has been less studied, and its localization in cells was currently uncertain (Table S1). This approach separated 396 BCa patients into two risk groups, with the median score as the cutoff. The risk model was validated further using a PCA distribution 3D plot, which verified perfect separation between distinct risk sample sets (Figures 3(c)–3(e)). The low-risk group outlived the high-risk group in terms of disease-specific and progression-free survival (Figures 3(f)–3(m)).

### 3.3. Verification of Eight Signature OSlncRNAs

**Figure 4:**
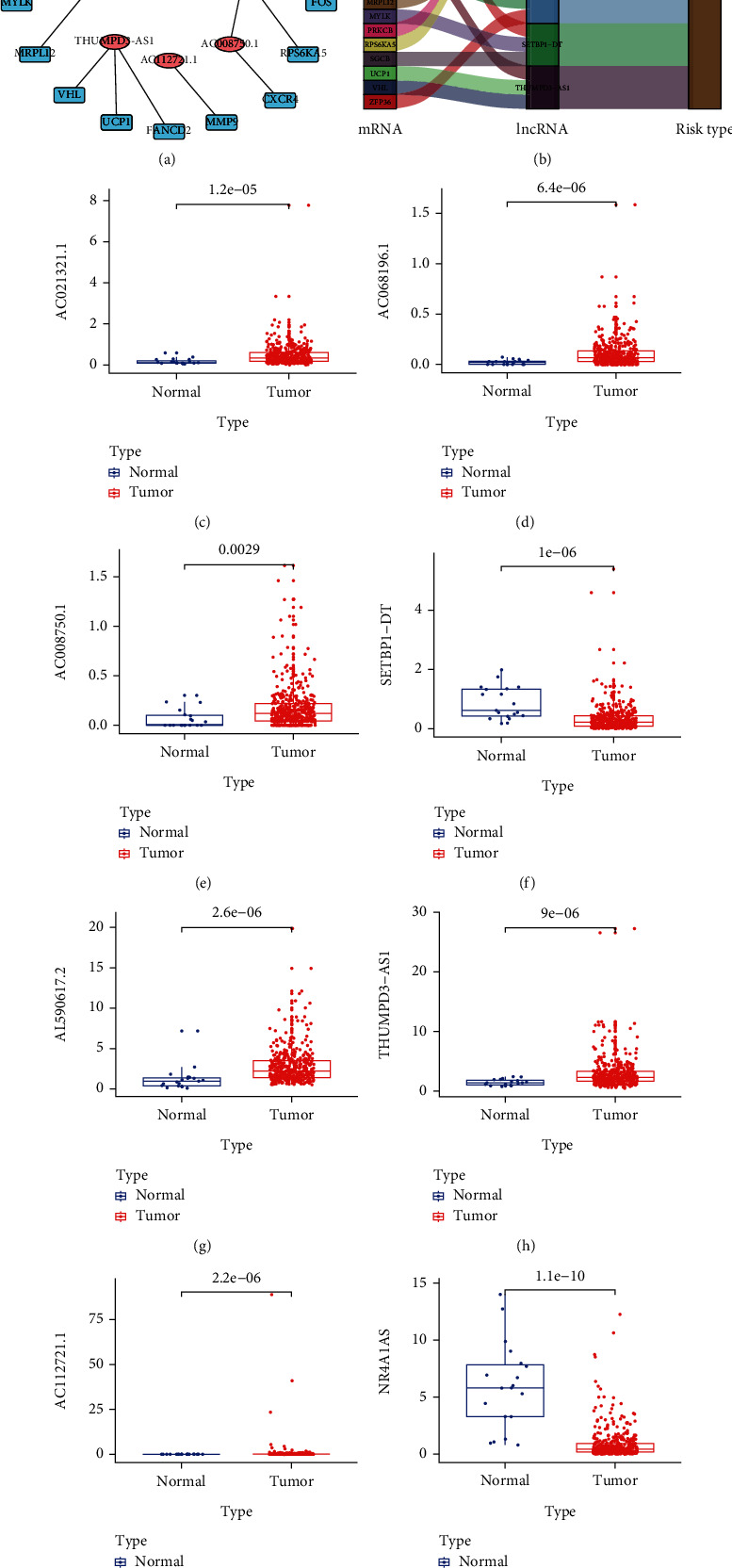
The variability of the eight DEOSlncRNAs. (a) The network between eighteen differential oxidative stress genes and eight DEOSlncRNAs. (b) Sankey plot between eighteen differential oxidative stress genes and eight DEOSlncRNAs. Box plot of the variability of the eight DEOSlncRNAs: (c),AC021321.1, (d) AC068196.1, (e) AC008750.1, (f) SETBP1-DT, (g) AL590617.2, (h) THUMPD3-AS1, (i) AC112721.1, and (j) NR4A1AS.

The relationship between the eighteen correlated DEOSGs and eight DEOSlncRNAs is shown in Figures 4(a) and 4(b). The expression levels of eight DEOSlncRNAs in the normal and tumor groups from the BCa dataset in TCGA are shown in Figures 4(c)–4(j)).

**Figure 5:**
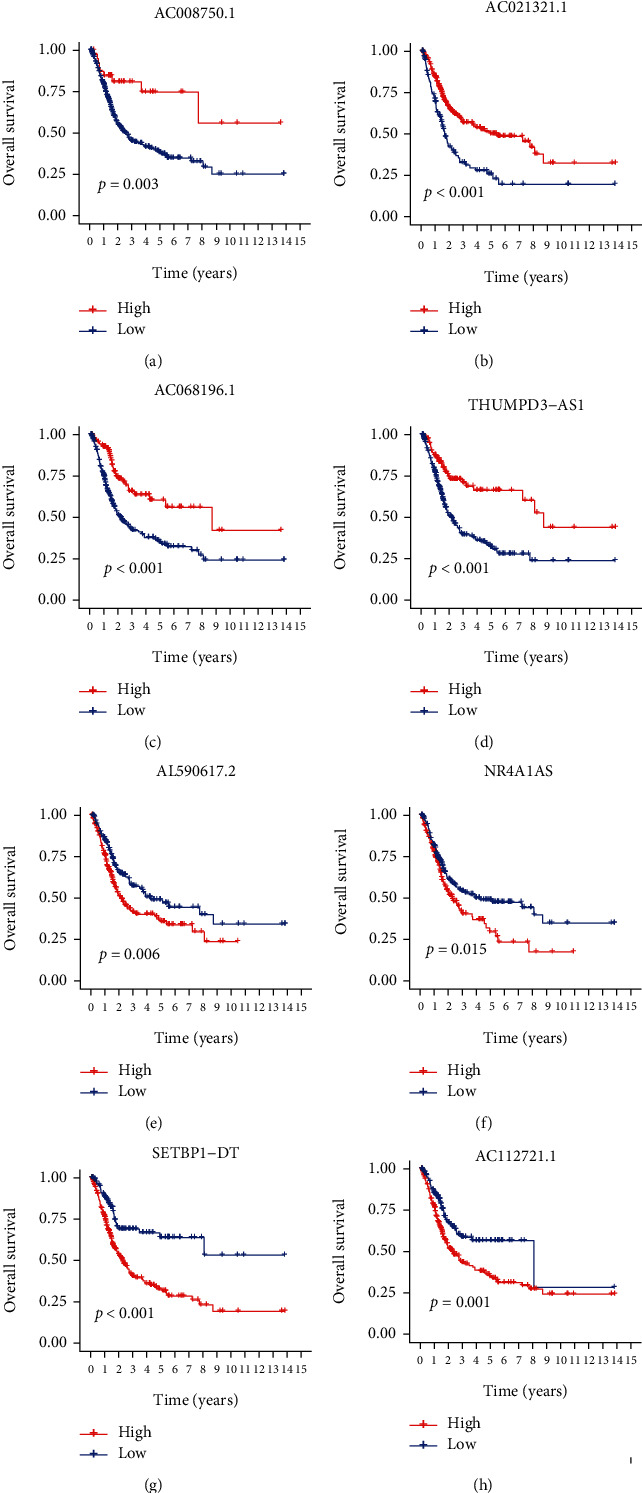
Prognostic values of the eight DEOSlncRNAs in the high- and low-risk groups. (a) AC008750.1, (b) AC021321.1, (c) AC068196.1, (d) THUMPD3-AS1, (e) AL590617.2, (f) NR4A1AS, (g) SETBP1-DT, and (h) AC112721.1.

To determine the prognostic association of eight hub lncRNAs, we analyzed the prognosis of eight hub lncRNAs in the context of the clinical information of BCa patients. The results indicated that overall survival rates were increased in patients with a high expression of AC008750.1, AC021321.1, AC068196.1, and THUMPD3-AS1 (Figures 5(a)–5(d)) and a low expression of AL590617.2, NR4A1AS, SETBP1-DT, and AC112721.1 (Figures 5(e)–5(h)).

### 3.4. Validation of the Prognostic Signature in the Test Set and the Entire Set

**Figure 6:**
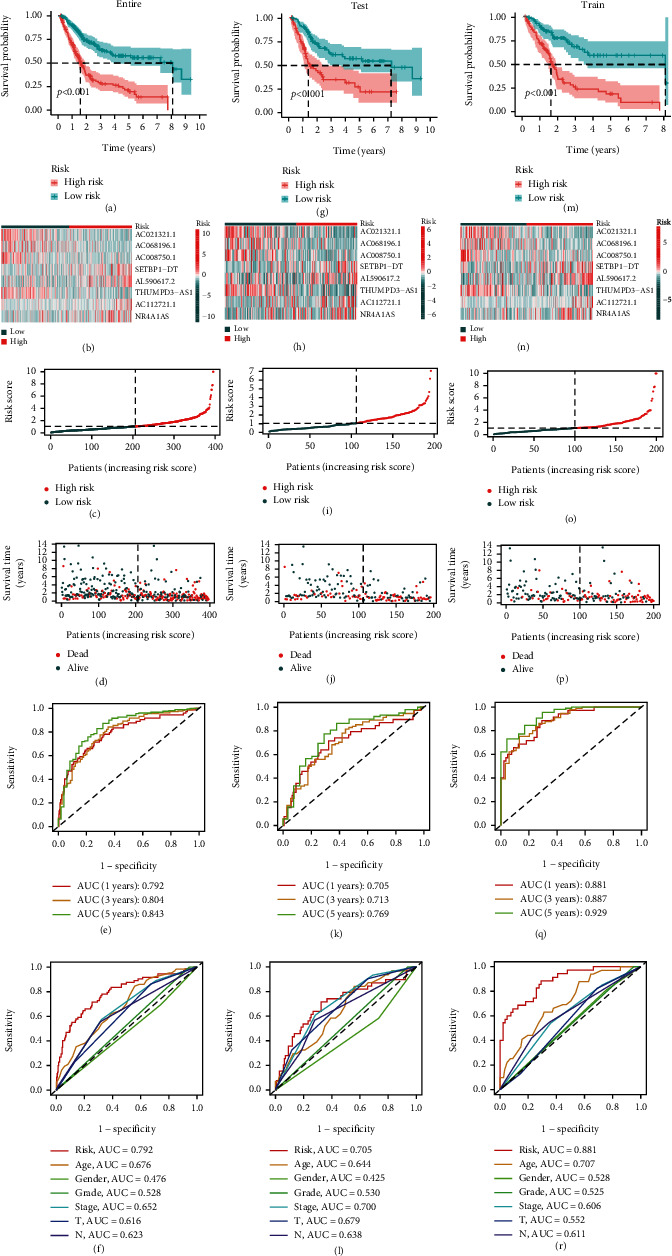
Construction and verification of the oxidative stress lncRNAs risk model of BCa patients. (a–f) The Kaplan–Meier curves of overall survival; heatmap; risk score; survival time; time-dependent ROC curves predicted 1-year, 3-year, and 5-year overall survival; and multivariate time-dependent ROC curve predicted the AUC for age, gender, grade, stage, T, and risk score of the total survival for 1-year survival for BCa patients in the TCGA entire cohort. (g–l) Test cohort. (m–r) Train cohort.

The prognostic signature's predictive value was evaluated in both the test set (*n* = 196) and the complete set (*n* = 396). In the test and whole sets, the risk score formula was used to assess the distribution of risk scores, survival status, survival time, and associated expression criteria of these lncRNAs in patients between the low-risk and high-risk groups. According to these findings, the high-risk group had a worse prognosis (Figures 6(a)–6(p)).

The sensitivity and specificity of the model for predicting prognosis were assessed using ROC curves. We further examined the ROC curve results by calculating the area under the ROC curve (AUC). The 1-, 3-, and 5-year AUCs were 0.792, 0.804, and 0.843, respectively, in the entire set; 0.705, 0.713, and 0.769, respectively, in the test set; and 0.881, 0.887, and 0.929, respectively, in the training set (Figures 6(e), 6(k), and 6(q)). The clinical variables and risk score had the strongest predictive capacity according to the risk model's 1-year ROC curve (Figures 6(f), 6(l), and 6(r)).

### 3.5. Risk Score and Clinical Molecular Subtypes

The conventional clinicopathological characteristics, namely, age, gender, grade, stage, M-stage, N-stage, and T-stage, were also consistent. We further explored a significant relationship between the risk score and clinicopathological characteristics; age; gender; tumor grade; M, N, and T-stages; and immunophenotyping. Our results showed that the risk scores for patients with stage IV, N0, T4, and C1 disease were significantly higher than those of patients with other disease stages. Meanwhile, the risk score was not significantly related to age, gender, grade, and M-stage (Figure S1A-H). We further found a significant decrease in stem cell content with an increasing risk score (Figure S1I).

Survival curves indicated that patients in the high-risk group with age, gender, high-grade, M0, N0-1, T3-4, and stages III-IV disease had a poorer prognosis, and low-grade M1 and T0-2 disease was not significantly correlated with prognosis, indicating the good predictive accuracy of this model (Figure S2A-N).

### 3.6. Construction of the Nomogram

**Figure 7:**
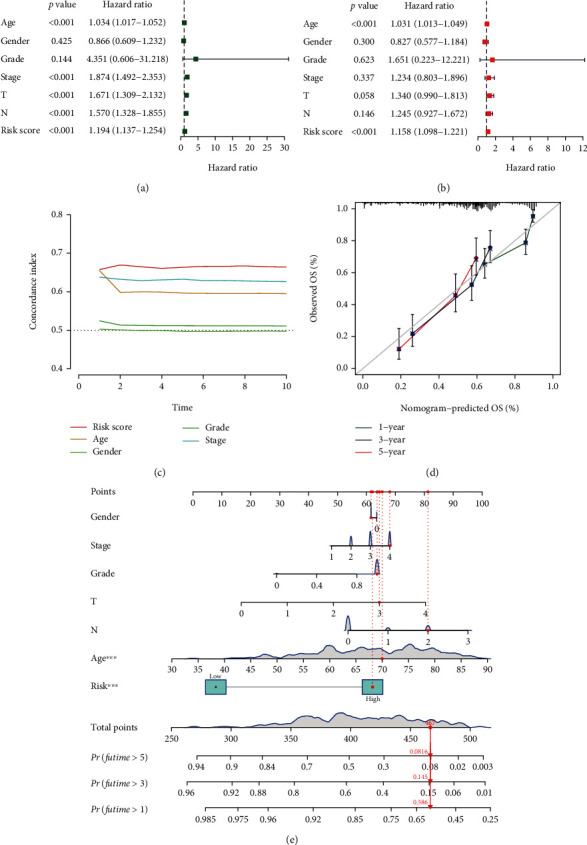
Nomogram and assessment of the risk model. (a and b) Univariate and multivariate Cox regression analyses of clinical factors (age, gender, grade, stage, T, and N) and risk score with overall survival. (c) Concordance index of risk score and clinical characteristics (age, gender, grade, and stage). (d) The calibration curves for 1-, 3-, and 5-year overall survival. (e) The nomogram combined risk scores and clinical characteristics (gender, stage, T, N, and age) to forecast the probability of 1-, 3-, and 5-year overall survival. ^∗∗∗^*p* < 0.001.

According to univariate Cox regression, the risk score hazard ratio and 95 percent confidence interval (CI) were 1.194 and 1.137-1.254 (*p* < 0.001), respectively, and 1.158 and 1.098-1.221 (*p* < 0.001), respectively, according to multivariate Cox regression (Figures 7(a) and 7(b)). Furthermore, age was also an independent prognostic parameter (1.031 and 1.013–1.049; *p* < 0.001) (Figure 7(b)). The concordance index of the risk score was the highest, indicating that the risk score is more accurate in predicting the prognostic outcome than other clinical information (Figure 7(c)).

We also utilized 1-, 3-, and 5-year calibration plots to confirm that the nomogram was in good agreement with the prediction of overall survival (Figure 7(d)). Based on independent prognostic factors, namely, risk score, age, T-stage, N-stage, stage, gender, and grade, we constructed a nomogram for predicting the 1-, 3-, and 5-year overall survival incidences of BCa patients (Figure 7(e)).

### 3.7. GO and KEGG Enrichment Analysis of the Two Risk Groups

We performed GO and KEGG analysis of differentially expressed genes in the low-risk and high-risk groups to better understand the underlying biological processes. The differentially expressed genes were predominantly enriched in BCa-related biological processes through the results of the GO analysis, such as “keratinocyte differentiation,” “keratinization,” “extracellular matrix disassembly,” “epidermal cell differentiation,” and “epidermis development” (Figure S3A-B). According to the KEGG analysis, we found that these differentially expressed genes were significantly enriched in “ECM − receptor interaction,” “proteoglycans in cancer,” “focal adhesion,” “IL−17 signaling pathway,” “protein digestion and absorption,” “renin−angiotensin system,” “Amoebiasis,” and “PI3K−Akt signaling pathway” (Figure S4A-B). We constructed a heatmap containing thirty highly expressed genes in the high-risk group and 30 highly expressed genes in the low-risk group (Figure S8).

### 3.8. Tumor Mutational Burden Analysis in the Risk Model

**Figure 8:**
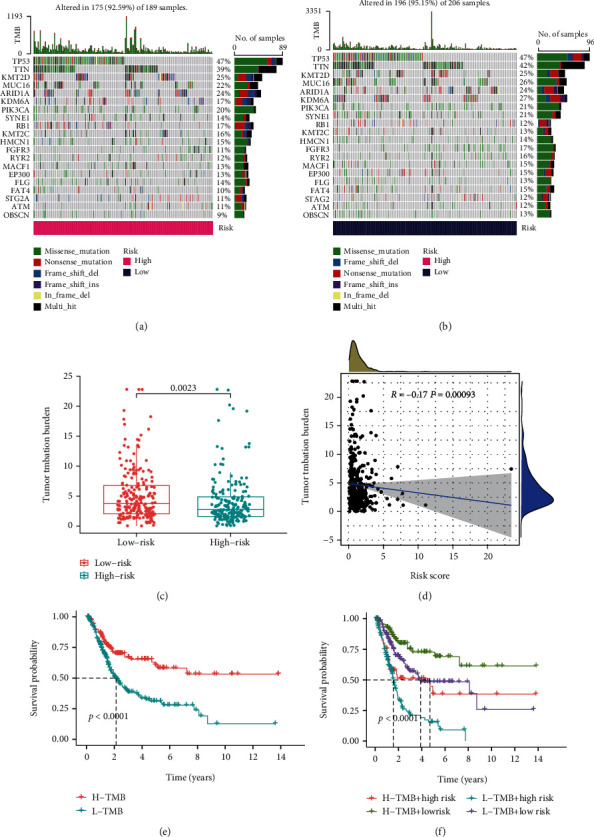
Differences and prognosis in TMB. (a) The waterfall plot and heatmap of mutation genes in the high-risk group. (b) The waterfall plot and heatmap of mutation genes in the low-risk group. (c) Boxplots showing TMB scores in different risk groups. (d) Correlation between TMB score and risk score. (e) Survival curves of the H-TMB group and the L-TMB group. (f) Survival curves of H-TMB and L-TMB scores in the different risk groups.

The waterfall plots indicated that the top mutated genes between the two risk groups were TP53 and TTN after BCa patients were split into high-TMB (*n* = 189) and low-TMB (*n* = 206) groups based on the median value of the TMB score. Overall, comparing the most frequent somatic mutations between the two groups, the profiles of TP53 and TTN were similar between the two groups concerning their mutation frequencies (Figures 8(a) and 8(b)). The mutation frequency of KDM6A was 27% in the low-risk group, while it was <20% in the high-risk group. Further study found that the TMB scores of patients in the low-risk group were typically higher than those in the high-risk group (Figure 8(c)). TMB decreased significantly with increasing risk score (*p* < 0.05) (Figure 8(d)). According to the Kaplan–Meier results, a high TMB increased patient survival compared to a low TMB (Figure 8(e)). Interestingly, various groups of patients with varying TMB scores showed diverse prognoses in this research. In the Kaplan–Meier analysis, patients in the low-risk category with a high TMB had a considerably better prognosis than those in the other categories (Figure 8(f)).

### 3.9. Immune Function Analysis and Immunotherapy

We explored differences in immune function between the two groups by calculating ESTIMATE, immune, and stroma scores for BCa samples. The study's results found that patients in the high-risk group scored significantly higher in all three categories than those in the low-risk group (Figure S5A-C). Further investigation of the distribution of immune cells in BCa showed that the low-risk group was enriched with large numbers of naive B cells, plasma cells, CD8 T cells and Tregs. In contrast, the high-risk group contained a higher proportion of resting memory CD4 T cells and neutrophils (Figure S5D-F). To further explore the correlation between immune cell infiltration and risk score, we found that the risk score was inversely correlated with the function of CD8 T cells and Tregs but positively correlated with the function of M0 macrophages neutrophils and M2 macrophages (Figure S6B-F). Tumor immune dysfunction and exclusion (TIDE) scores were significantly higher in the high-risk group than in the low-risk group (Figure S5G).

However, with the growth of the notion of precision medicine in recent years, immune checkpoint and inhibitor (ICI) treatment has attracted extensive attention [33]. As a result, more studies on the various amounts of immune checkpoint expression in different groups will serve to give benchmarks for precision medicine. Especially, the total expression levels of genes, including TNFSF9, CD44, PDCD1LG2, CD200, and NRP1, were significantly greater in the high-risk group, while ADORA2A, TNFRSF25, TNFRSF14, CD40LG, LGALS9, TNFRSF15, CD160, and TMIGD2 were higher in the low-risk group (Figure S6A). Furthermore, when the clinical treatment value was considered, the high-risk group was more responsive to A.443654, A.770041, AICAR, AUY922, AZ628, AMG.706, AG.014699, and AZD.0530 than the low-risk group, and the low-risk group was more sensitive to ABT.263, AKT.INHIBITOR VIII, AXITINIB, ATRA, and ABT.888 (Figure S7A-M).

## 4. Discussion

BCa is one of the most prevalent urological malignant tumors. The main clinical treatments currently include surgical tumor resection and adjuvant chemotherapy. However, the prognosis of patients with BCa has not improved in recent years due to tumor recurrence and drug resistance [34]. There were currently few approaches for predicting OS-related lncRNAs in BCa patients. Our findings identified eight lncRNAs linked to OS. NK cells were activated in vitro to induce AC008750.1 expression to generate antitumor capacity against lung adenocarcinoma [35]. AC112721.1 expression was higher in breast cancer and BCa than that in normal tissue [36, 37]. AL590617.2 expression increased with the progression of prostate cancer grade. It may form a complex with MYC to transcriptionally regulate MYC targets or affect tumor progression through MYC recruitment to transactivate genes such as MARVELD1, HOXB7, PYCR3, AMIGO2, BNIP3L, and ZNF121 [38]. NR4A1AS was upregulated in oral squamous cell carcinoma and promoted the proliferation of oral squamous cell carcinoma cells by upregulating miR-221 through demethylation [39]. In two other articles, similar results to those of the present study were found in the prognostic model of BCa constructed by THUMPD3-AS1 [40, 41]. SETBP1-DT, AC068196.1, and AC021321.1 were currently less well studied and not be described specifically.

In this research, we focused on OS-related lncRNAs and pathways by investigating the correlation between gene expression and gene mutations. We collected clinical data from BCa patients in TCGA to confirm that the risk model DEOSlncRNA had good prognostic significance. In addition, based on DEOSlncRNAs, eight of them (AC021321.1, AC068196.1, AC008750.1, SETBP1-DT, AL590617.2, THUMPD3-AS1, AC112721.1, and NR4A1AS) were selected by LASSO regression to build a risk score model, which demonstrated that patients with higher risk scores were more likely to encounter adverse outcomes compared with patients with lower risk scores. TMB analysis showed significant differences in the mutant genes KDM6A and TTN and prognosis in different groups, and their gene functions need to be further investigated. Further studies of the immune microenvironment showed a higher proportion of resting memory CD4 T cells and neutrophil phenotypes in patients in the higher-risk group with higher immune scores.

In contrast, patients in the low-risk group with lower immune scores had a higher proportion of naïve B cells, plasma cells, CD8 T cells, and Tregs phenotypes. Interestingly, both patients in the low-risk group and those in the high-risk group were sensitive to multiple chemotherapeutic agents. Immune checkpoint analysis showed significant differences in 16 genes (ADORA2A, TNFSF9, TNFRSF25, CD44, PDCD1LG2, TNFRSF14, BTNL2, CD40LG, CD200, LGALS9, IDO2, NRP1, TNFSF15, CD160, TMIGD2, and HHLA2) based on this risk score. Among the immune checkpoints, CD44 could promote cell-cell and cell-matrix interactions, proliferation, differentiation, invasion, and migration and is a cell adhesion receptor mainly expressed in tumors and tumor stem cells. ROS levels in cancer cells were often reduced by the coupling of some CD44 variants to the glutamate-cysteine transporter XCT, making cancer cells resistant to chemotherapy and radiotherapy [42]. Intestinal epithelial cells could ablate the ECM and tight junctions by triggering OS [43]. Neutrophils could inhibit the production of IL-17 in the tumor microenvironment by inducing OS, thus exerting an antitumor effect [44]. Regarding the renin-angiotensin system, previous literature has shown that angiotensin II induces OS in prostate cancer and promotes inflammation [45]. The PI3K-Akt signaling pathway was a classical OS pathway associated with cell growth and differentiation [46].

Tumor mutational load was an important biological marker indicative of tumor mutational status and has been considered an effective method for discovering potential tumor immune regulatory pathways [22, 47]. Notably, in the present investigation, the prognostic survival analysis of L-TMB was worse than that of H-TMB, and patients in the high-risk group had lower survival rates with either L-TMB or H-TMB compared with the low-risk group, which indicated that patients in the high-risk group may most urgently need targeted therapy or combination therapy.

The function of immune cell types was affected by OS, which directly or indirectly induced tumor development and became an obstacle to immunotherapy [48]. Long-noncoding RNAs associated with scorch death revealed that risk scores were positively correlated with M0 and M2 macrophages and negatively correlated with prognosis [49]. Thus, these studies indicated that different immune cell subpopulations strongly affected cancer progression, which further demonstrated the accuracy of predictive models based on DEOSlncRNA constructs.

IC50 was the half-inhibitory concentration, which reflected the patient's sensitivity to the drug in this article. The lower the IC50 values, the stronger the sensitivity. In A.443654, A.770041, AICAR, AUY922, AZ628, AMG.706, AG.014699, and AZD.0530, the IC50 value of the high-risk group was lower than that of the low-risk group, so the sensitivity of the high-risk group was higher than that of the low-risk group. Similarly, in ABT.263, AKT.INHIBITOR VIII, AXITINIB, ATRA, and ABT.888, the sensitivity of the low-risk group was higher than that of the high-risk group. This also provided a new basis for the individualized treatment of BCa patients.

Currently, risk score models are mostly built using LASSO regression methods. The ROC curve indicated that this risk model had higher sensitivity and specificity than other clinical indicators in predicting the prognosis of BCa. At the same time, patients with different risk scores showed different prognostic outcomes in BCa clinical subgroups, and the correlation between risk scores and clinical index analysis showed that immune scores were statistically different in N-stage, T-stage, and grade of BCa (Figure S1). In T-stage, we found that immune scores increased with increasing T-stage and were statistically significant, suggesting that NMIBC was associated with lower immune scores. In comparison, MIBC was associated with higher immune scores. This result indicated that an increased risk score was associated with invasive or metastatic BCa and was consistent with previous prognostic results (Figure 6). This had reference significance for the early diagnosis and personalized medicine of BCa patients. The development of targeted drugs based on risk models may improve the treatment effect of NMIBC and MIBC. Immune checkpoint inhibitors were a potential cancer treatment that blocks key molecules and showed excellent anticancer efficacy, particularly in revolutionizing the clinical progression of metastatic and locally advanced BCa [50, 51].

In conclusion, our research has several limitations. First, we used R and statistical analysis to develop prognostic risk models for eight DEOSlncRNAs using public databases. Although these approaches have been used and proved in numerous studies [52, 53] and some of the eight DEOSlncRNAs have been studied to some extent [35–41], further research is needed to reveal the association between eight DEOSlncRNAs and OS. And more, in-depth studies are needed including their functions and molecular mechanisms. Second, we have internally validated the risk model in BCa transcriptome data downloaded in TCGA, and more sequencing data will be needed for external validation in the future. Third, we collected the clinical sample information of BCa patients in public databases to construct the model, which laid a foundation for future clinical applications. However, more clinical sample information is required to increase the model's credibility.

## 5. Conclusions

In conclusion, we have screened eight DEOSlncRNAs that were utilized to conduct a risk model. This risk model can predict the prognosis and immune status of BCa patients and effectively differentiate NMIBC and MIBC, thus providing favorable treatment options for patients with BCa.

## Figures and Tables

**Table 1:** Eight DEOSlncRNAs with BCa in the TCGA dataset were identified by LASSO analysis.

Gene	HR	Lower 95% CI	Upper 95% CI	Coefficient	*p* value
AC021321.1	0.285311974	0.125446119	0.648907459	-1.02655	0.002776
AC068196.1	0.113629968	0.015911989	0.811449103	-1.80103	0.030138
AC008750.1	0.205638247	0.064731943	0.65326463	-2.01385	0.00732
SETBP1-DT	1.85386739	1.102382313	3.117633744	0.734441	0.019939
AL590617.2	1.571522901	1.139970992	2.1664448	0.613165	0.005785
THUMPD3-AS1	0.585684206	0.397611657	0.862716127	-0.33666	0.006784
AC112721.1	1.330182562	1.103569024	1.603330293	0.335326	0.002752
NR4A1AS	1.368998896	1.008550835	1.858268232	0.3252	0.043949

## Data Availability

The data are available at the TCGA database (https://tcga-data.nci.nih.gov/tcga/), the GeneCards database (https://www.genecards.org/), and the DIANA Tools (https://diana.e-ce.uth.gr/lncbasev3).

## Supplementary Materials

Supplementary Materials Table S1: the detailed information of eight DEOSlncRNAs for constructing the risk model. Figure S1: the correlations of risk score with clinical features. Figure S2: the clinical utility of the risk score. Figure S3: GO enrichment analysis. Figure S4: KEGG enrichment analysis. Figure S5: tumor-infiltrating immune cells of BCa patients. Figure S6: checkpoints and immune cells. Figure S7: the immunotherapy prediction of risk groups. Figure S8: heatmap of differential genes between the high-risk and low-risk groups.
