# Supplementary material for: A Novel Risk Model for lncRNAs Associated with Oxidative Stress Predicts Prognosis of Bladder Cancer
Source: J Oncol. 2022 Oct 11;2022:8408328. doi: 10.1155/2022/8408328 (PMC9578793; doi:10.1155/2022/8408328)
Supplement: Supplementary Materials — Table S1: the detailed information of eight DEOSlncRNAs for constructing the risk model. Figure S1: the correlations of risk score with clinical features. Figure S2: the clinical utility of the risk score. Figure S3: GO enrichment analysis. Figure S4: KEGG enrichment analysis. Figure S5: tumor-infiltrating immune cells of BCa patients. Figure S6: checkpoints and immune cells. Figure S7: the immunotherapy prediction of risk groups. Figure S8: heatmap of differential genes between the high-risk and low-risk groups. [file 8408328.f1.docx]

Table S1 The detailed information of eight DEOSlncRNAs for constructing the risk model.

| **Gene** | **Location** | **Transcript size** | **Subcellular localization** |
| --- | --- | --- | --- |
| AC021321.1 | chr8:68,256,210-68,257,322 | 1113 bp | Nucleus |
| AC068196.1 | chr2:181,940,778-181,970,695 | 833 bp | unknow |
| AC008750.1 | chr19:51,414,298-51,414,965 | 492 bp | Nucleus |
| SETBP1-DT | chr18:44,676,927-44,679,717 | 2065 bp | Nucleus |
| AL590617.2 | chr6:139,015,456-139,018,164 | 421 bp | Nucleus |
| THUMPD3-AS1 | chr3:9,426,948-9,440,263 | 1115 bp | Cytoplasm/Nucleus |
| AC112721.1 | chr2:238,330,063-238,333,919 | 588 bp | Nucleus |
| NR4A1AS | chr12:52,058,459-52,059,503 | 687 bp | Nucleus |

Figure S1


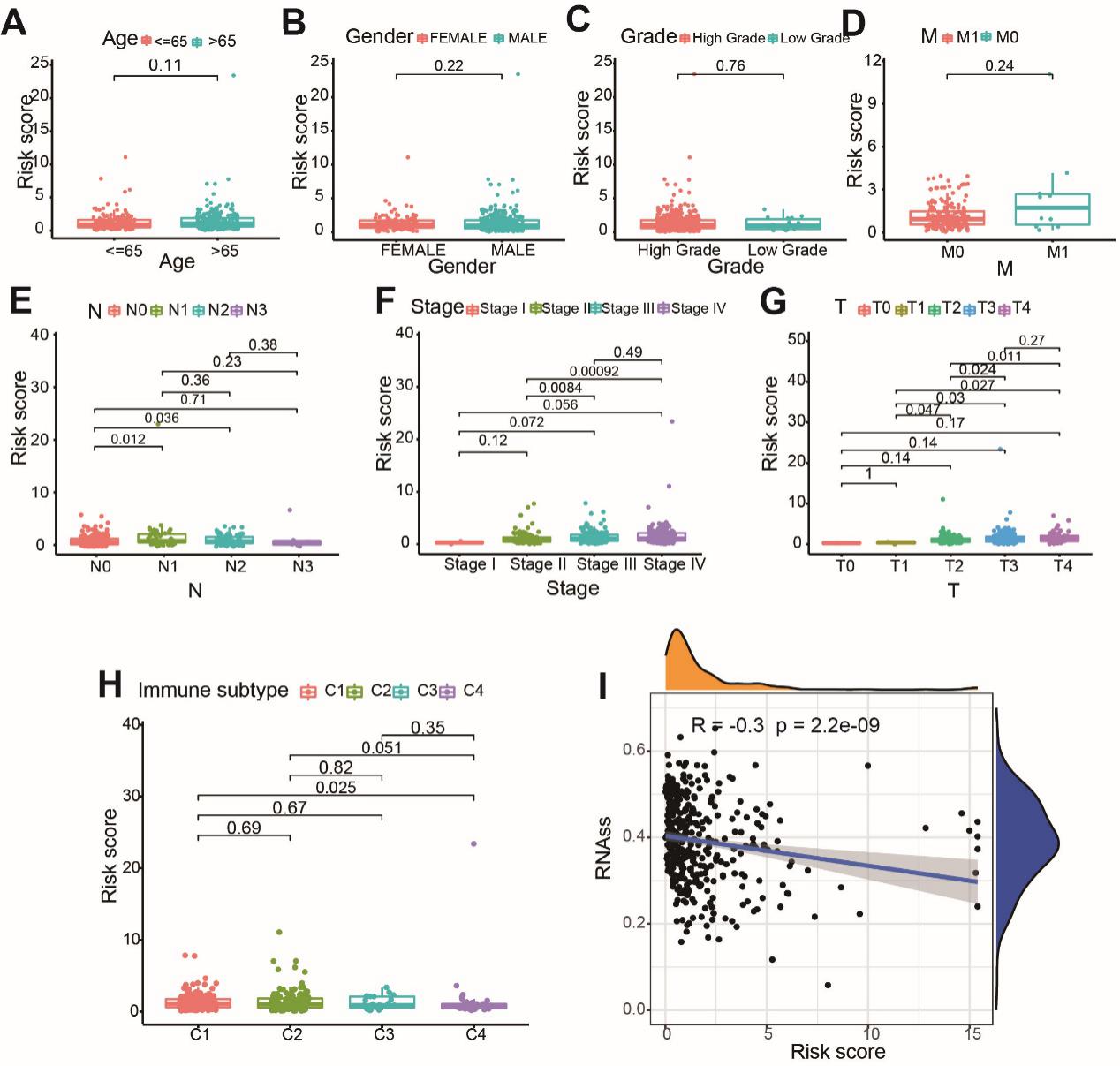


Figure S1. The correlations of risk score with clinical features. (A) Age (B) Gender (C) Grade (D) M-stage (E) N-stage (F) Stage (G) T-stage (H) Immune subtype (I) RNAss

Figure S2


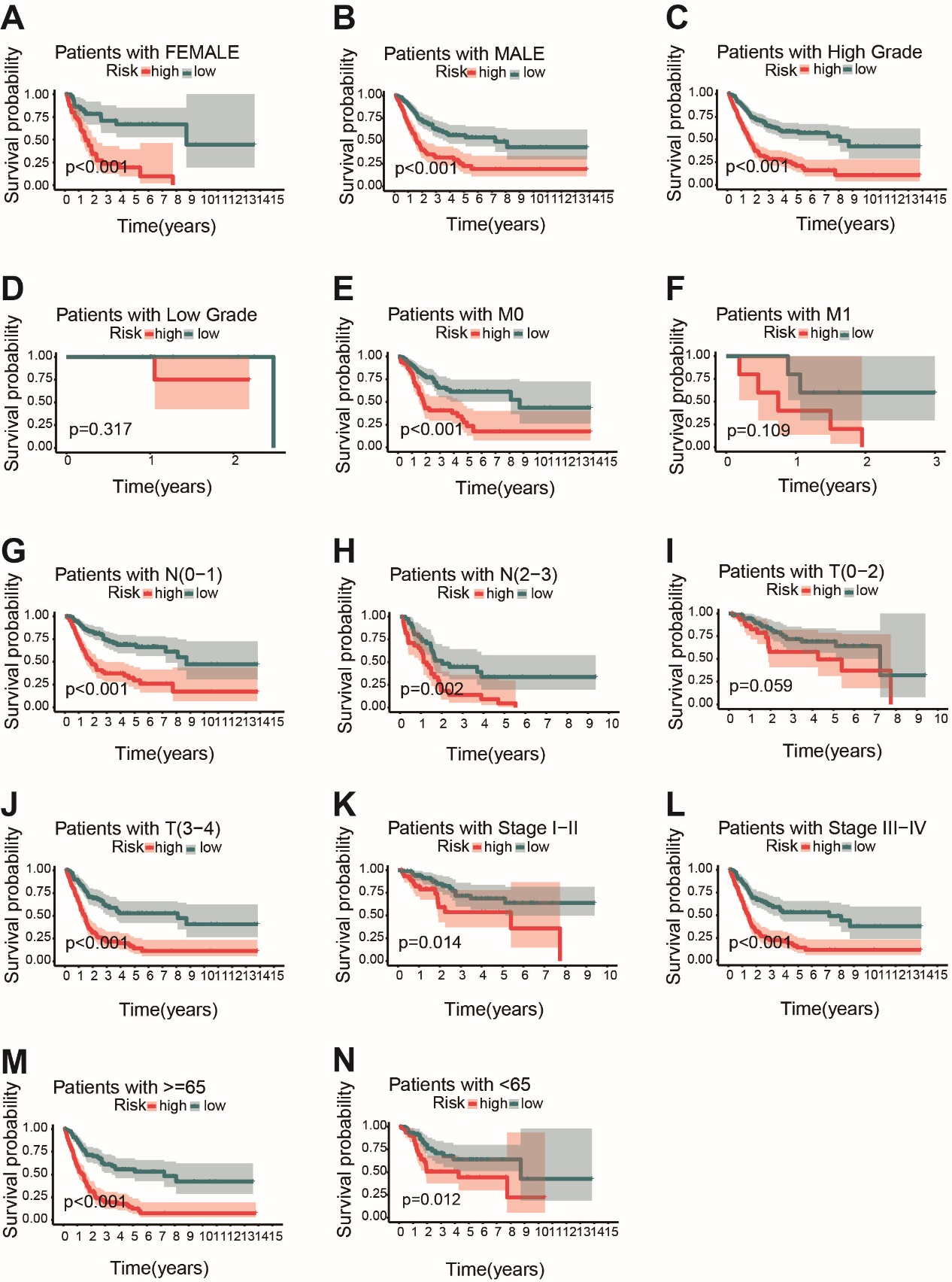


Figure S2. The clinical utility of the risk score. (A-B) Gender. (C-D) Grader. (E-F) M-stage. (G-H) N-stage. (I-J) T-stage. (K-L) Stage. (M-N) Age.

Figure S3


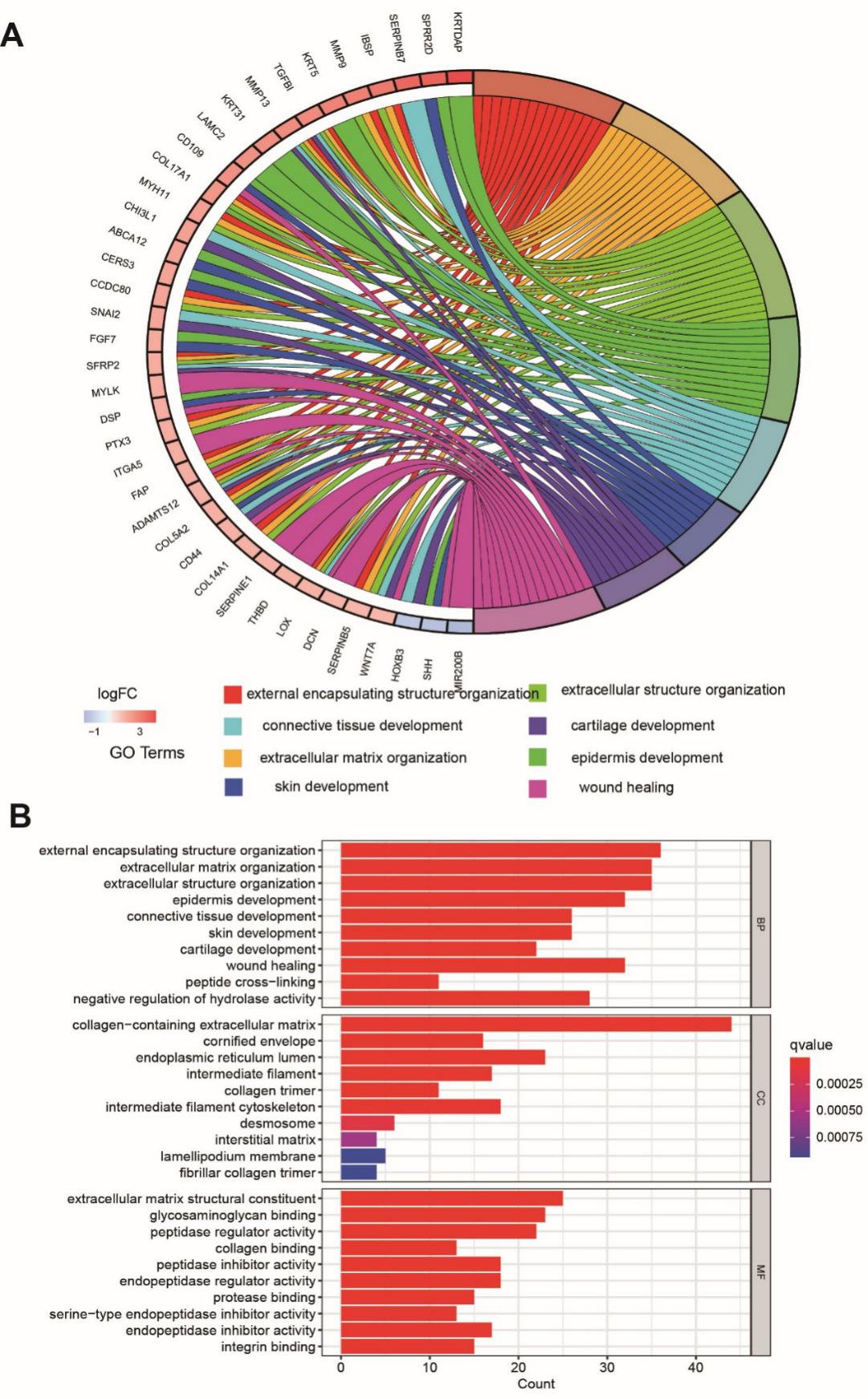


Figure S3. GO enrichment analysis. (A-B) GO enrichment analysis in the differentially expressed genes between the low-risk and high-risk groups.

Figure S4


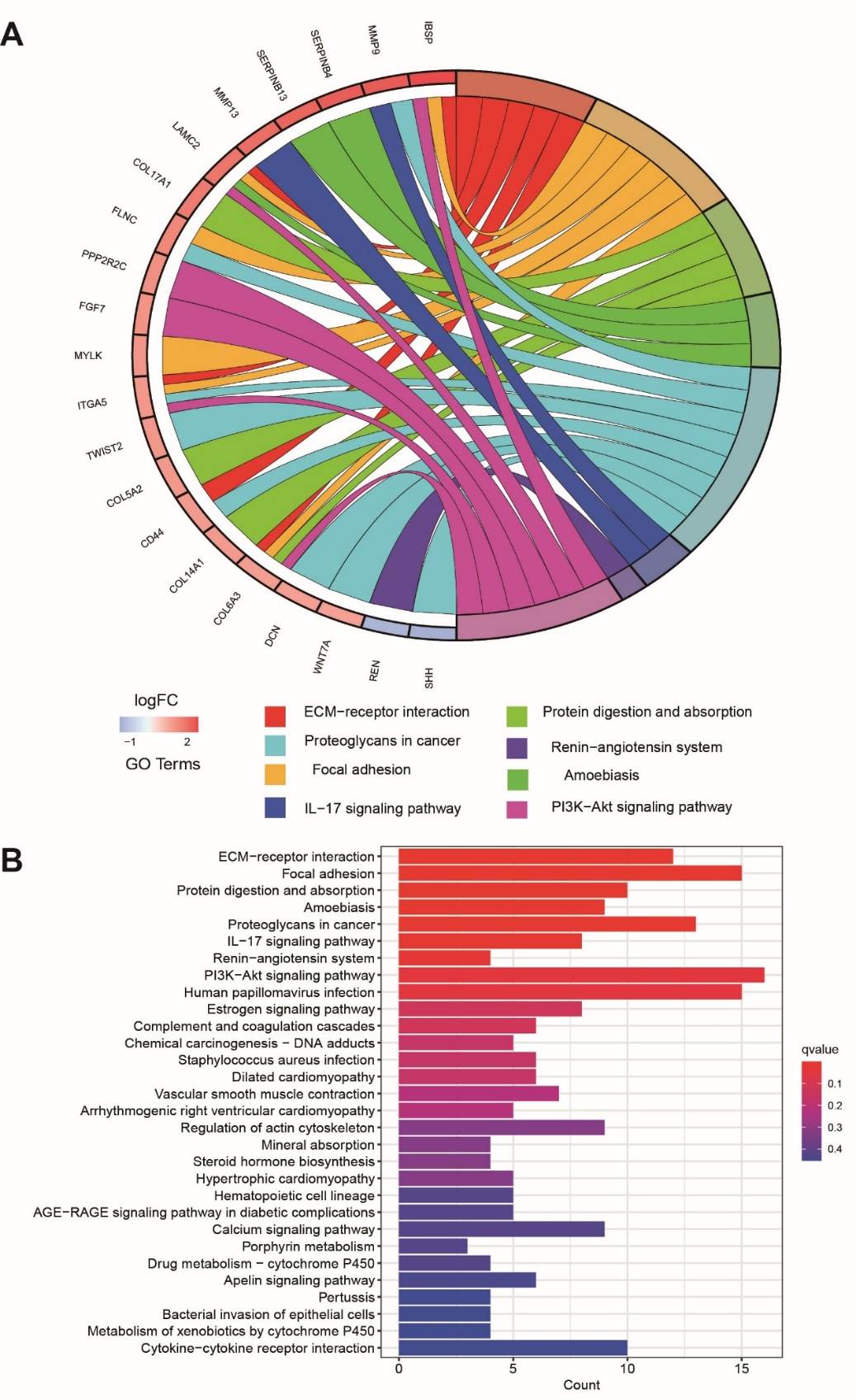


Figure S4. KEGG pathway enrichment analysis. (A-B) KEGG pathway analysis in the differentially expressed genes between the low-risk and high-risk groups.

Figure S5


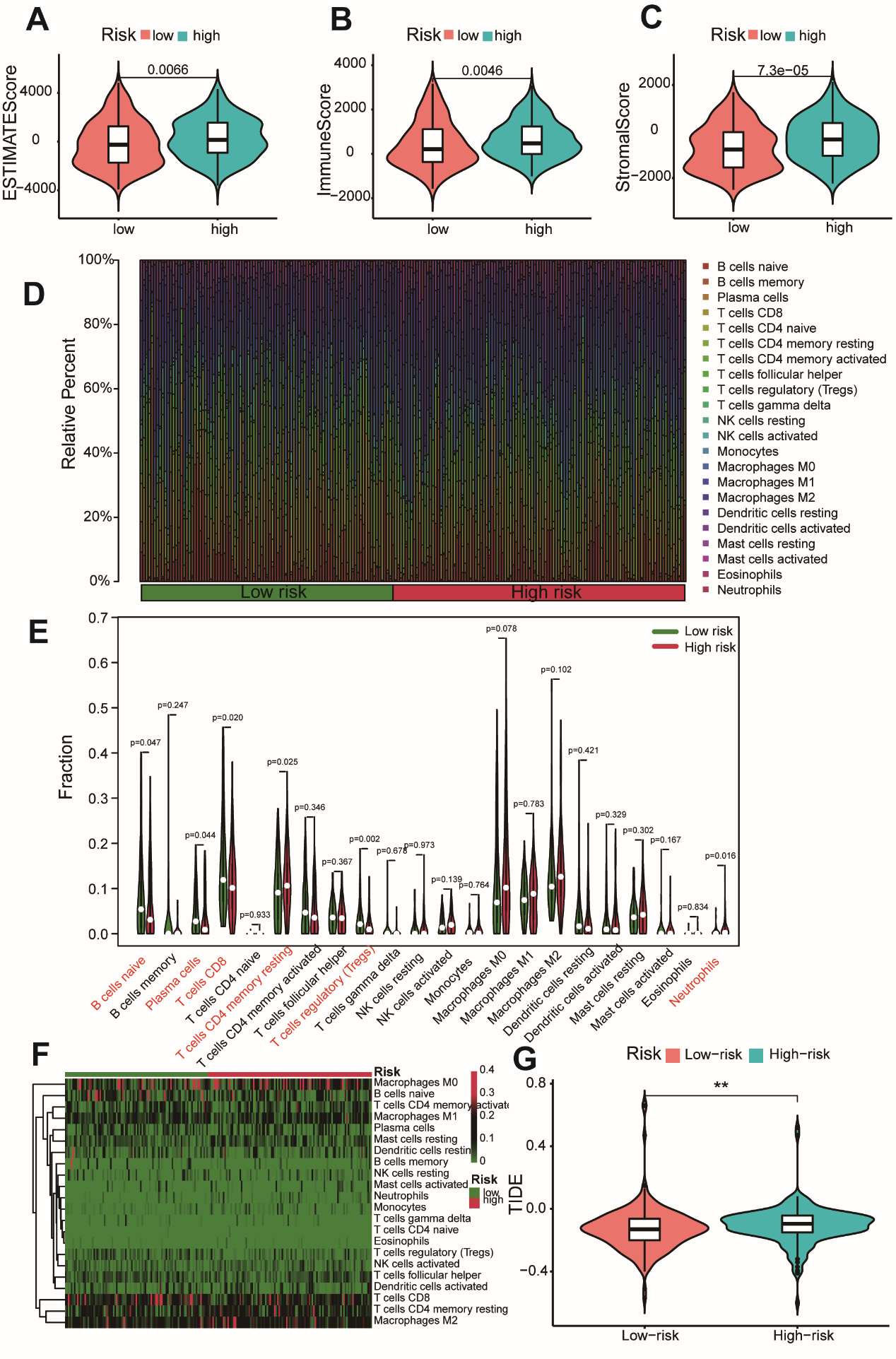


Figure S5. Tumor-infiltrating immune cells of BCa patients. (A-C) Estimate score, immune score, and stromal score in low-risk and high-risk groups. (D) The proportions of different tumor-infiltrating immune cells in the low-risk and high-risk groups. (E) Violin plot showed the different proportions of tumor-infiltrating cells between the high-risk and low-risk groups. (F) Heatmap showed the different proportions of tumor-infiltrating cells between the high-risk and low-risk groups. (G) The boxplot representation of TIDE scores in high-risk and low-risk groups. ***P* < 0.01.

Figure S6


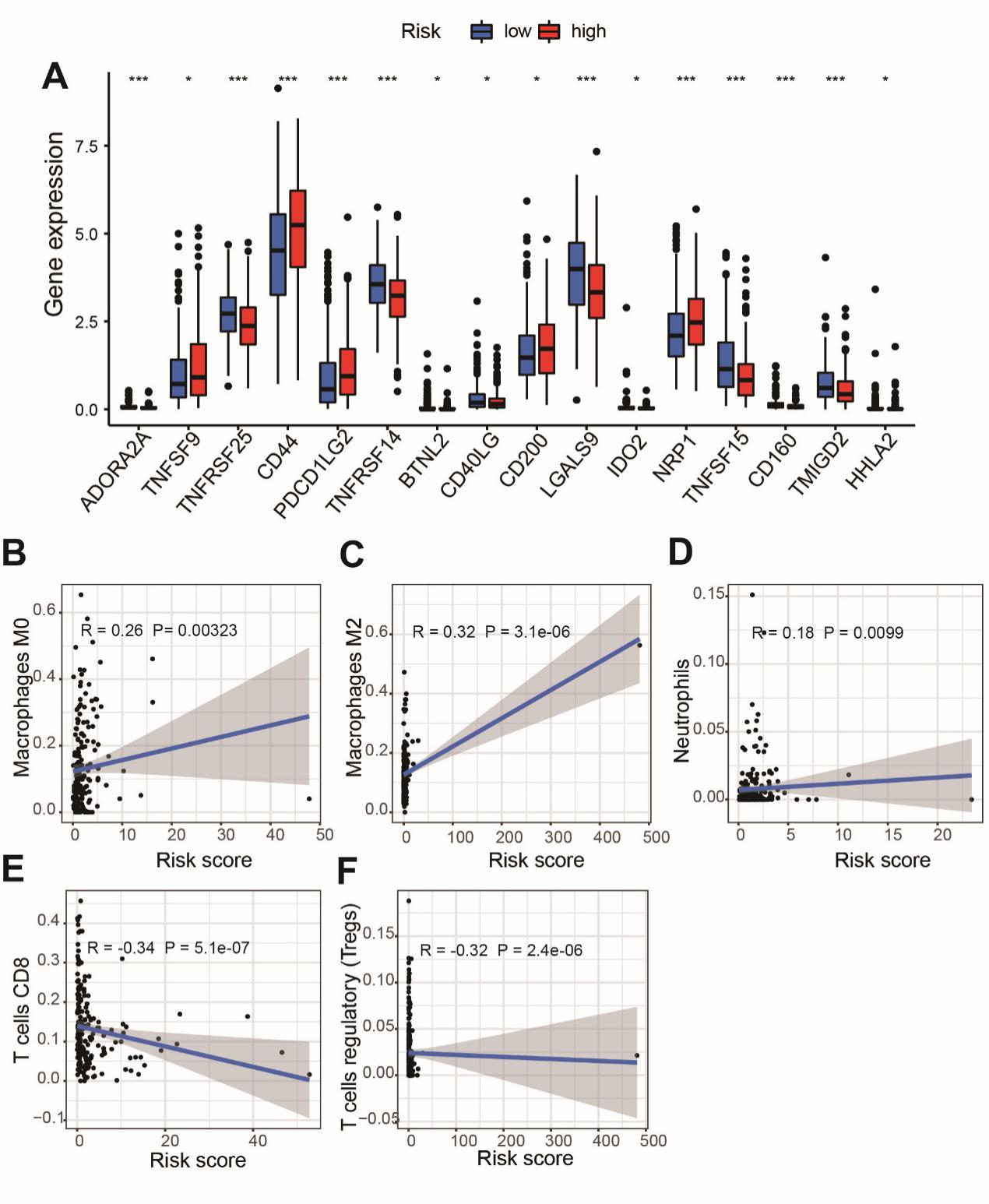


Figure S6. Immune checkpoints and immune cells. (A) The difference of 15 immune checkpoints expression in risk groups. (B-F) The correlation between risk score and immune cells. **P* < 0.05, ***P* < 0.01, ****P* < 0.001.

Figure S7


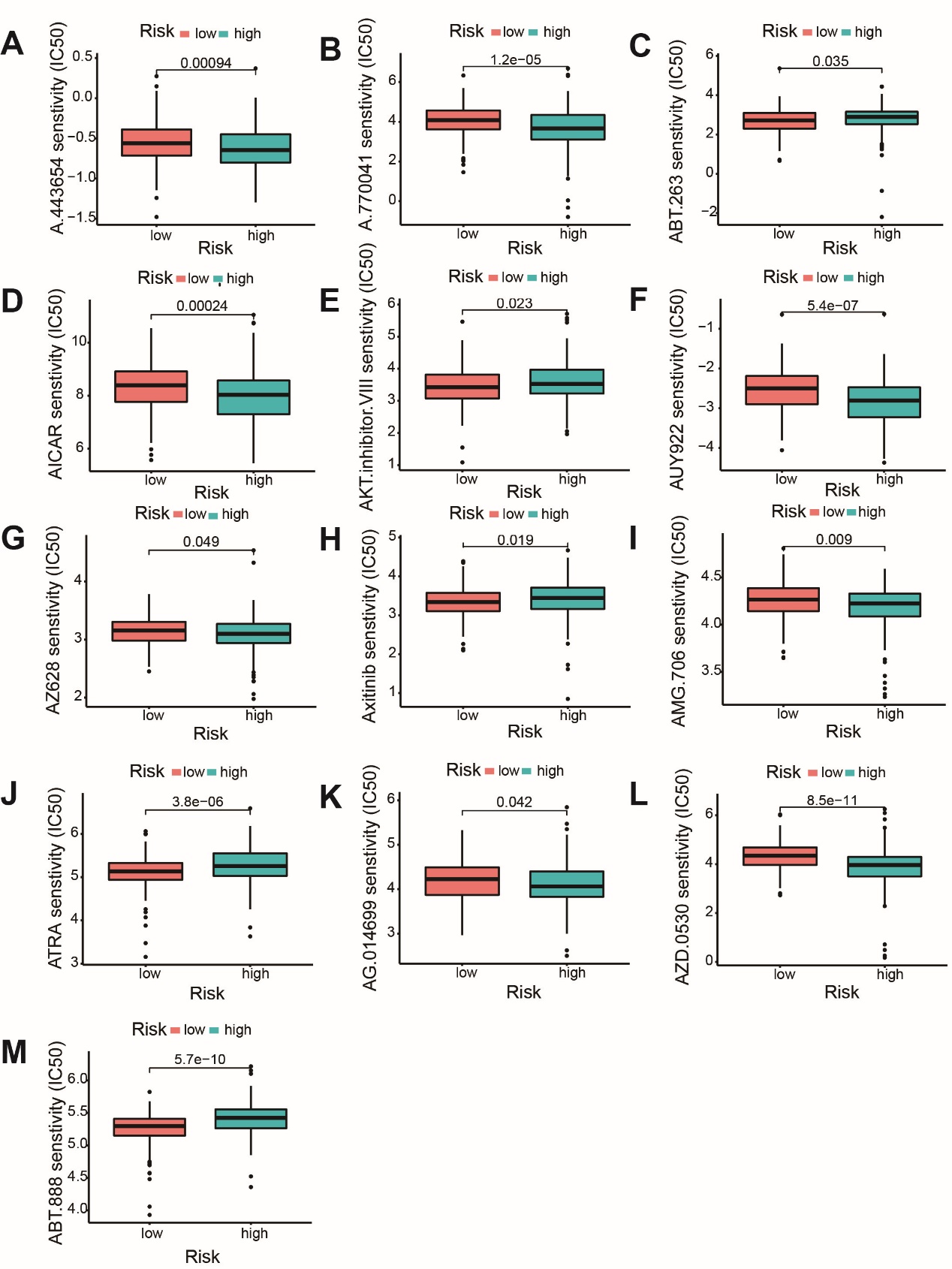


Figure S7. The Immunotherapy prediction of risk groups. Thirteen immunotherapeutic drugs solely showed a significant IC50 difference in low-risk and high-risk groups. (A) A.443654 (B) A.770041 (C) ABT.263 (D) AICAR (E) AKT.inhibitor.VIII (F) AUY922 (G) AZ628 (H) Axitinib (I) AMG.706 (J) ATRA (K) AG.014699 (L) AZD.0530 (M) ABT.888

Figure S8


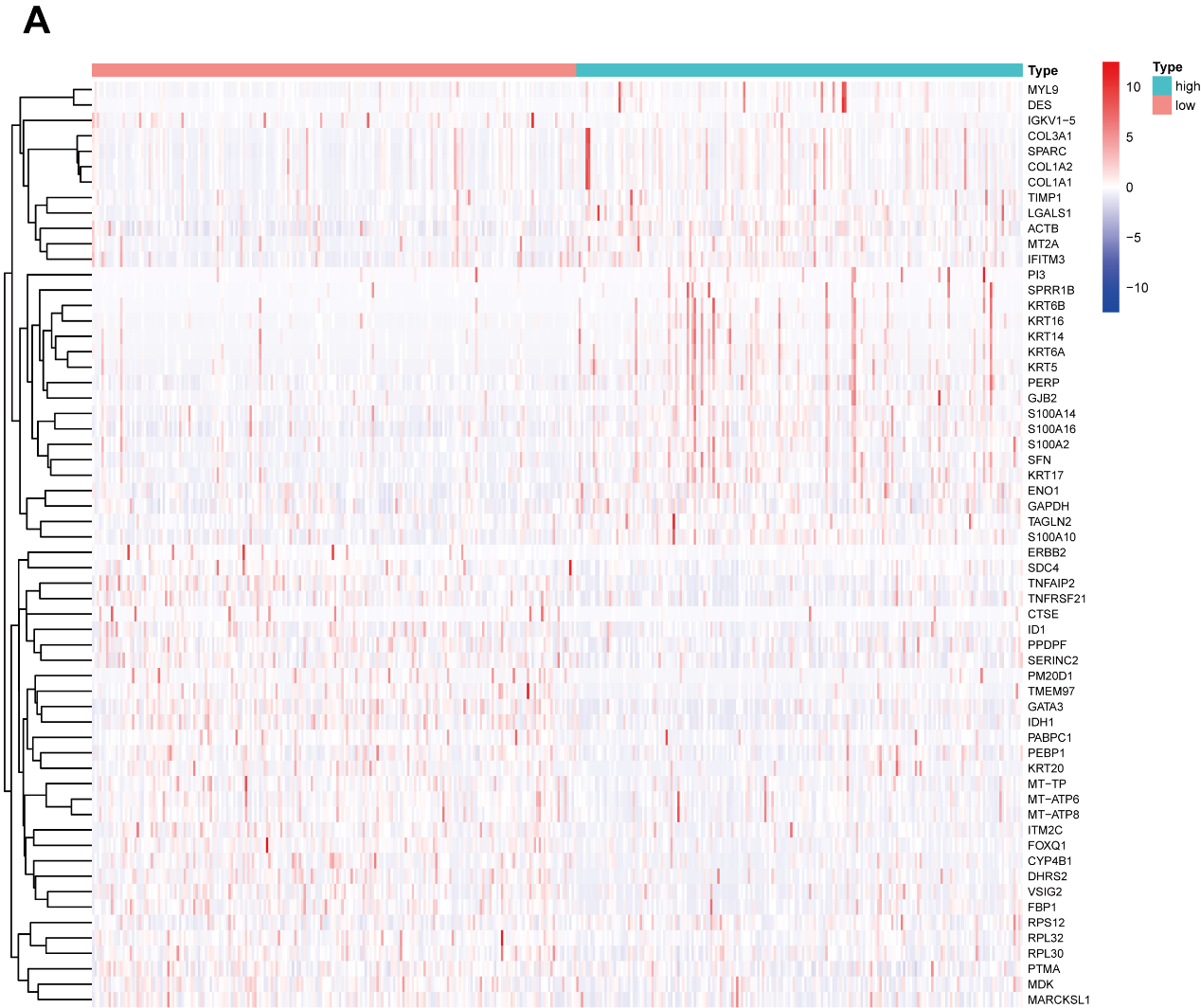


Figure S8. Heatmap of differential genes between high-risk and low-risk groups. (A)Heatmap of 30 genes that are highly expressed in the high-risk group and 30 genes that are highly expressed in the low-risk group.
